# Optimizing Timing of Peripheral Blood Stem Cell Collection: A Comparative Study of Day-4 Versus Day-5 Post-G-CSF Mobilization in Healthy Allogeneic Donors

**DOI:** 10.3390/biomedicines14081824

**Published:** 2026-08-13

**Authors:** Dania Sarhan, Sakhr Alshwayyat, Rama Amin Al-Wreidat, Heba Alya, Zaid Abdel Rahman, Mohammad Ma’koseh, Maysa Al-Hussaini

**Affiliations:** 1 Department of Cell Therapy and Applied Genomics, King Hussein Cancer Centre, Amman 11941, Jordan; dsarhan@khcc.jo (D.S.); ra.17235@khcc.jo (R.A.A.-W.); ha.10859@khcc.jo (H.A.); 2 Department of Surgery, King Hussein Cancer Centre, Amman 11941, Jordan; sa.17557@khcc.jo; 3 BMT Program, King Hussein Cancer Centre, Amman 11941, Jordan; za.11040@khcc.jo (Z.A.R.); mm.09744@khcc.jo (M.M.)

**Keywords:** allogeneic stem cell transplantation, apheresis, CD34+ cell, granulocyte colony-stimulating factor, peripheral blood stem cell

## Abstract

**Background and objectives:** At King Hussein Cancer Center, peripheral blood stem cell (PBSC) collections in healthy allogeneic donors are typically initiated on day 4 of granulocyte colony-stimulating factor (G-CSF) mobilization, contingent upon circulating CD34+ cell counts (≥20 CD34+/µL). In cases where counts are suboptimal, collections are typically deferred to day 5. **Research method:** We conducted a retrospective cohort study on healthy allogeneic donors who underwent G-CSF mobilization and PBSC collection at KHCC between 2019 and 2023, comparing CD34+ dose on days 4 and 5 post G-CSF administration, to optimize collection timing, maximize yield, improve donor safety and institutional efficiency. The minimum cell dose considered acceptable for collection was set at 2.5 × 10^6^ CD34+/kg. Continuous variables were summarized using the sample median and range. Categorical variables were summarized as numbers and percentages. CD34+ counts from days 4 and 5 were analyzed using a paired t-test. Statistical analysis was conducted using SPSS (version 29). **Result and findings:** We identified 526 donors during the study period: 326 (62%) donors collected on day 4 only, and 44 (8.4%) donors collected on day 5 only. This study focused on the remaining 156 (29.7%) donors who underwent collections on both days. For this group, the median age was 23 years (range 2–67 years), with 50.9% males. Median circulating CD34+/µL was lower on day 4: 48 (7–162) compared to day 5 was 75 (13–215). Mean cell dose collected (CD34+/kg) was significantly lower on day 4 compared to day 5 (4.4 × 10^6^ vs. 6.1 × 10^6^, respectively, *p* < 0.001), with 76% and 94% achieving an adequate collection dose on either day alone. In donors with a circulating CD34+ count <20 CD34+/µL on day 4, the likelihood of achieving an adequate cell dose was 30%, compared to 74% when the circulating CD34+ count was ≥20 CD34+/µL (*p* < 0.009). **Conclusions:** Peripheral CD34+ count on day 4 is a reliable predictor of stem cell yield. Delaying collection to day 5 in donors with suboptimal circulating CD34+ cells significantly improves yield and minimizing associated costs. These findings support evidence-based timing of collection to optimize donor outcomes and institutional efficiency in allogeneic PBSC mobilization.

## 1. Introduction

Allogeneic hematopoietic stem cell transplantation is an established curative therapy for a broad range of hematological malignancies and bone marrow failure syndromes, with approximately 55,000–60,000 procedures performed worldwide each year [1]. Granulocyte colony-stimulating factor mobilized peripheral blood stem cells have largely displaced bone marrow as the dominant graft source, and now account for roughly 75% of all allogeneic procedures [1,2]. The infused CD34+ cell dose remains the principal determinant of post-transplant outcomes. Doses above 4.5 × 10^6^/kg recipient body weight have been associated with faster neutrophil and platelet engraftment, as well as lower non-relapse mortality, in both myeloablative and reduced-intensity conditioning regimens [3].

Therefore, contemporary transplant programs target a minimum of 4 × 10^6^ CD34+ cells/kg, and several centers have adopted a stricter target of 5 × 10^6^/kg in the post-pandemic period to offset the viability loss associated with cryopreservation. This shift reflects a marked change in graft handling, as fewer than 2% of allogeneic grafts were cryopreserved before March 2020 compared to over 80% during the early pandemic period [4,5]. These recipient-facing thresholds translate into a corresponding demand on the donor, as approximately 2–5% of healthy donors fail to mobilize an adequate dose, and a much larger fraction requires multiple apheresis sessions [6,7].

The same competing demands on the recipient and donor have motivated interest in collecting on day four rather than day five of mobilization, which would spare one day of G-CSF exposure and shorten the apheresis window. The published experiences remain divided. Recent series using continuous-flow platforms have reported adequate single-session day-four yields in over 90% of donors [8,9], whereas modeling work on the same platforms has projected success rates as low as 22.6% with standard once-daily G-CSF [10]. The peripheral blood CD34+ count is the most consistently reported laboratory predictor of yield [2], and most centers now apply a hybrid rule whereby collection proceeds on day four if a pre-specified CD34+ threshold is met and is otherwise deferred to day five [8]. However, the performance of this rule in practice, including its discriminative accuracy, the within-donor difference between day-four and day-five yields, and the consequences for product disposition, has not been characterized in a single-center cohort large enough for paired analysis.

Therefore, we reviewed consecutive G-CSF mobilized peripheral blood stem cell collections from healthy allogeneic donors at our institution to evaluate the day-four peripheral blood CD34+ triage rule, focusing on its discriminative accuracy, the within-donor difference between day-four and day-five yields, and the disposition of collected products as a downstream consequence of the triage decision.

## 2. Methods

### 2.1. Study Population and Design

This was a single-center, retrospective cohort study of consecutive G-CSF mobilized PBSC collections from healthy allogeneic donors at King Hussein Cancer Center between January 2019 and December 2023. The study was approved by the Institutional Review Board (25 KHCC 066).

Stem cell mobilization was performed using filgrastim (G-CSF) at a dose of 10 µg/kg/day, given as 5 µg/kg subcutaneously every 12 h.

**Figure 1:**
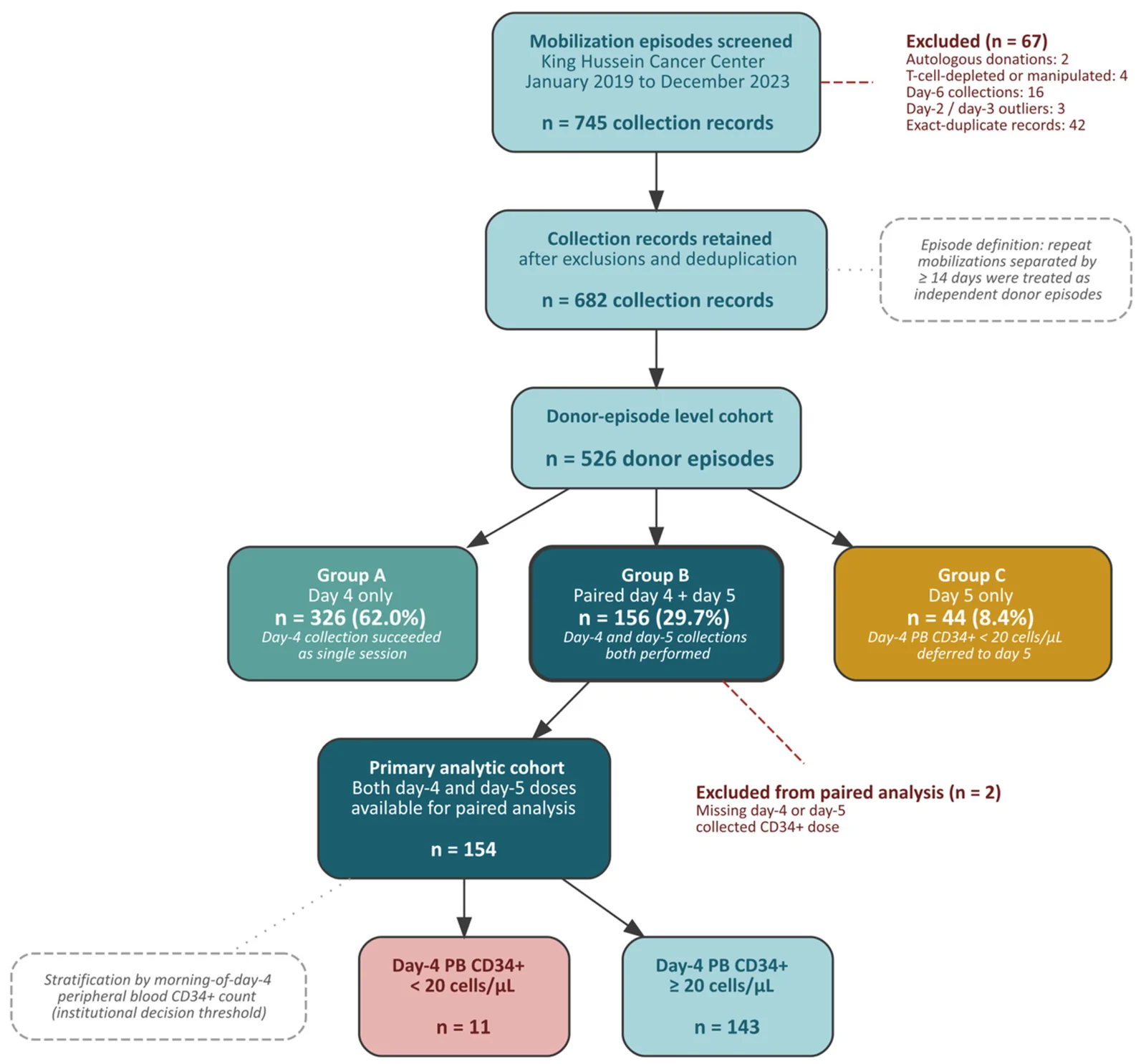
Cohort assembly, exclusion criteria, and group assignments.

Filgrastim was administered for four consecutive days, with two doses given daily (morning and evening). Peripheral blood CD34 cell counts were assessed on day 4 to evaluate mobilization status. If required, filgrastim administration was continued on day 5. Apheresis on day four proceeds when the morning peripheral blood CD34+ count is at or above 20 cells/µL and is otherwise deferred to day five. When the day-four collection fails to reach the institutional target of 2.5 × 10^6^ CD34+ cells/kg recipient body weight, a second apheresis is performed on day five. The average processed blood volume was 2.5 total blood volume (TBV) on day 4 and 2.2 TBV on day 5. Peripheral blood stem cell collection was performed via apheresis using the Spectra Optia machine. The cohort assembly, exclusions, and group assignments are summarized in Figure 1.

The primary outcome was the achievement of an adequate collection dose, defined as a collected CD34+ dose of at least 2.5 × 10^6^ cells/kg recipient body weight [3], assessed both on a single collection day and on the cumulative episode. The secondary outcomes were the within-donor difference between day-four and day-five CD34+ yield, discriminative performance of the day-four peripheral blood CD34+ count for predicting day-four dose adequacy, and disposition of collected products.

### 2.2. Statistical Analysis

Continuous variables were summarized as medians and interquartile ranges, and categorical variables as counts and percentages. Group comparisons were performed using the Kruskal–Wallis test for continuous variables and Pearson’s chi-square or Fisher’s exact test for categorical variables.

Day-four and day-five CD34+ yields were compared within paired donors using the Wilcoxon signed-rank test for the continuous dose and McNemar’s test for the binary dose-adequacy outcome. The within-donor median difference and 95% confidence interval were estimated using the Hodges–Lehmann estimator. Donors were then stratified by the institutional day-four threshold of 20 cells/µL, and stratum-specific proportions of dose adequacy were compared using Fisher’s exact test, with odds ratios and 95% confidence intervals reported.

The day-four peripheral blood CD34+ count was modeled as a continuous predictor of day-four dose adequacy using univariable logistic regression, and the predicted probability function was reported with its 95% confidence band. Discrimination was assessed using the area under the receiver operating characteristic curve with a 95% confidence interval. Sensitivity, specificity, positive predictive value, and negative predictive value were reported at the institutional threshold of 20 cells/µL and the threshold maximizing Youden’s J statistic.

To assess robustness, the paired day-four versus day-five comparisons were repeated using stricter dose definitions of 4 × 10^6^ and 5 × 10^6^ CD34+ cells/kg, corresponding to the EBMT recommendation and the post-pandemic target adopted by some centers. The operational impact was estimated as the proportion of paired donors whose day-four peripheral blood CD34+ count fell below the institutional threshold, together with the proportion of day-four products ultimately discarded.

All tests were two-sided, with significance set at *p* < 0.05, and confidence intervals were reported at the 95% level. Analyses were performed in R version 4.4 using the “pROC”, “flextable” and “officer” packages.

## 3. Results

### 3.1. Cohort Characteristics

The study cohort comprised 526 donor episodes after exclusion and deduplication. Of these, 326 (62.0%) were collected on day four only (Group A), 156 (29.7%) underwent both day-four and day-five apheresis (Group B), and 44 (8.4%) were deferred from day four and collected on day five only (Group C). The baseline donor and collection characteristics by group are summarized in Table 1. Day-four circulating CD34+ counts differed across groups, with the highest values in single-session donors and the lowest in donors who required two collections. The diagnostic distribution did not differ across the groups (*p* = 0.143).

### 3.2. Paired Yield Comparison

**Figure 2:**
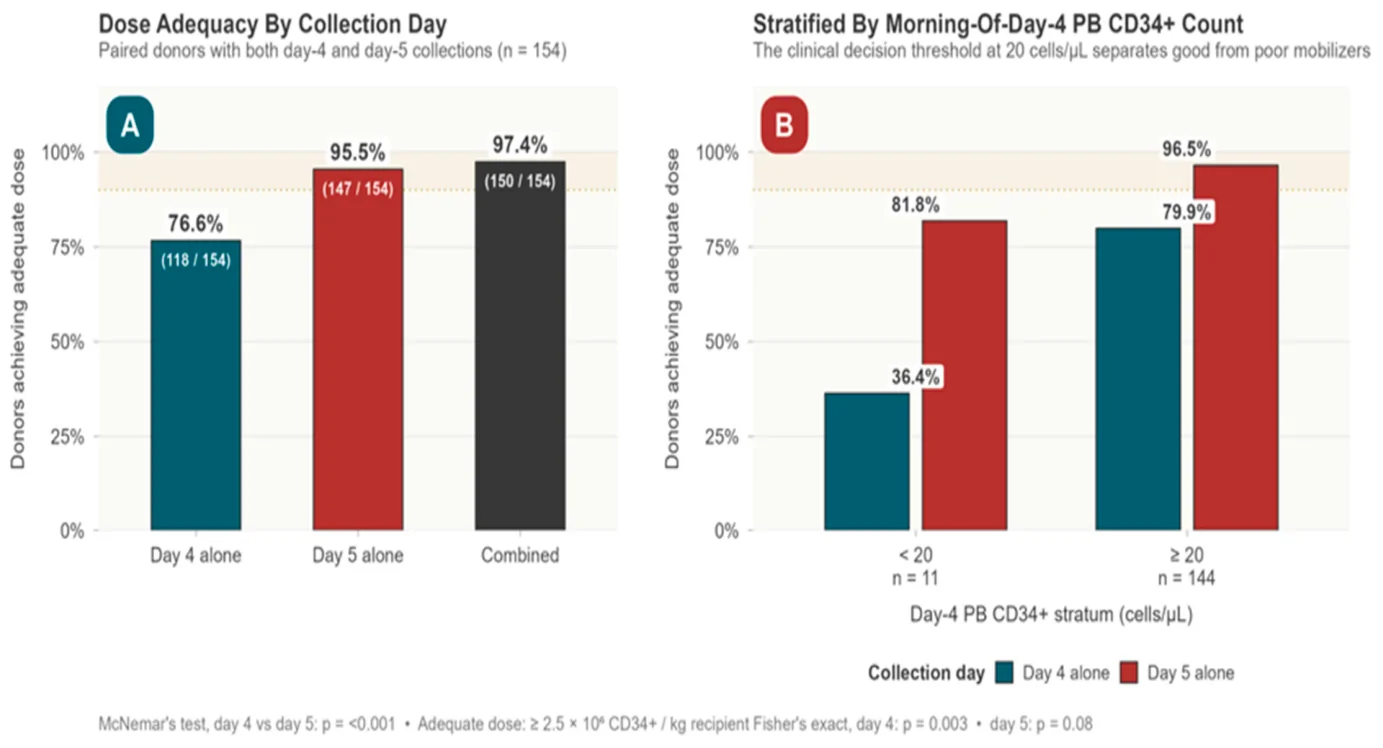
Paired comparisons of CD34+ cell yields between day-4 and day-5 collections. (**A**) Dose adequacy by collection day, (**B**) Dose adequacy by PB CD34+ count.

In the 154 donors with paired day-four and day-five CD34+ doses, day-five collection yielded a higher dose than day-four collection (Wilcoxon signed-rank test, *p* < 0.001), with a median within-donor difference of 1.54 × 10^6^ CD34+ cells/kg (95% CI 1.12 to 1.98) and a day-five to day-four median yield ratio of 1.42. The proportion of donors meeting the institutional adequate dose threshold of ≥2.5 × 10^6^ CD34+/kg was higher on day five than on day four (McNemar’s test, *p* < 0.001), and the same trend was observed at the stricter 4 × 10^6^ and 5 × 10^6^ thresholds (Supplementary Table S1). Three-quarters of the paired donors had a higher absolute yield on day five than on day four. The full paired comparison and stratified results are shown in Figure 2 and Table 2 and Table 3.

### 3.3. Discriminative Performance and Product Disposition

**Figure 3:**
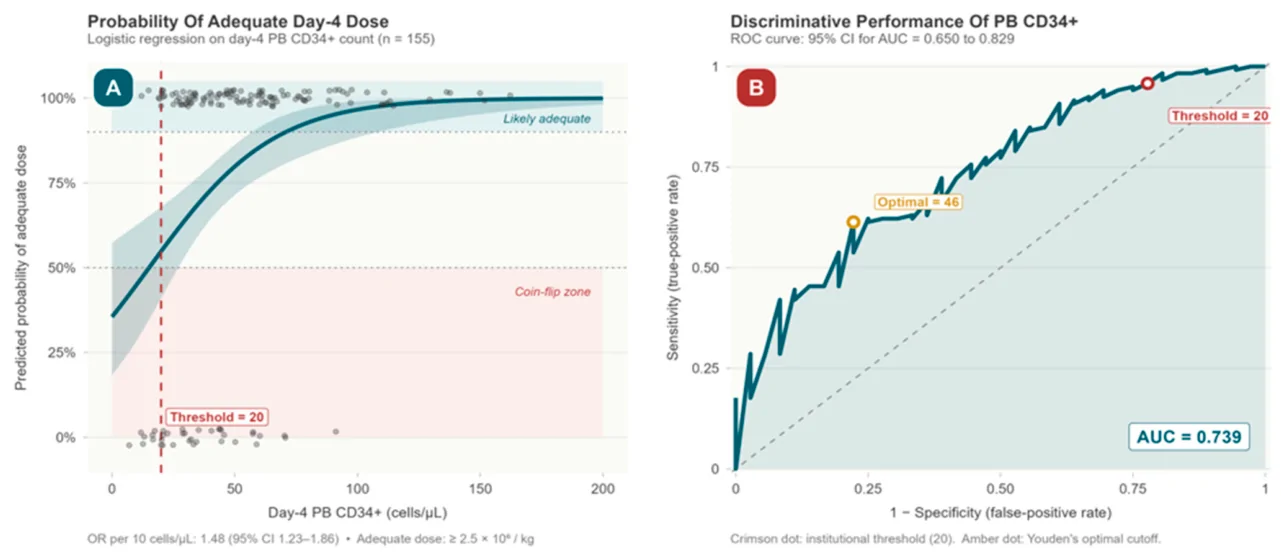
Discriminative performance and product disposition. (**A**) Probability of adequate Day-4 dose, (**B**) Discriminative performance of PB CD34+.

Day-four peripheral blood CD34+ count predicted day-four dose adequacy with moderate discrimination (Figure 3). The univariable logistic regression model showed an increasing probability of adequate day-four yield with increasing CD34+ count, and the area under the receiver operating characteristic curve was (0.739, 95% CI 0.650–0.829, n = 155). At the institutional threshold of 20 cells/µL, the sensitivity for an adequate day-four dose was 96.6%, and the specificity was 19.4%. In the paired cohort stratified at this threshold, donors with a day-four CD34+ cell count <20 cells/µL reached adequate day-four dose less than half as often as donors at or above the threshold (Fisher’s exact odds ratio 6.76, 95% CI 1.59 to 33.71). This estimate should be interpreted with caution, as the below-threshold stratum comprised only 11 donors, and the correspondingly wide confidence interval reflects its limited precision. After day-five collection, the adequacy proportions converged across the strata (Table 3).

Day-four products from paired donors (Group B) were less frequently used for clinical infusion than those from single-session donors (Group A) (Supplementary Figure S1, Supplementary Table S3). Among Group B day-four products with a known disposition at data lock, 19.1% were discarded, either fresh or after cryopreservation, compared with 8.6% in Group A. At data lock, a pending or unknown disposition was recorded for 41 of 156 (26.3%) day-four products in Group B and 14 of 326 (4.3%) in Group A (Supplementary Table S3); the higher pending fraction in Group B, reflecting donors not yet transplanted, means the 19.1% discard estimate rests on a smaller denominator and should be regarded as provisional. The remaining Group B day-four products were either pending clinical decision or stored as cryopreserved residual.

## 4. Discussion

Decisions regarding the timing of peripheral blood stem cell collection in healthy donors balance two obligations: delivering an adequate graft to the recipient and limiting donor exposure and procedural burden. Collecting on day four spares a day of G-CSF and an apheresis session for donors who succeed but commits those who fall short to a second collection attempt. Routine deferral to day five removes this risk at the cost of an additional day of G-CSF for the majority of donors from whom cells could be collected earlier. The manner in which a given rule navigates this trade-off is best understood through its operational characteristics rather than a single yield comparison.

The within-donor difference between day-four and day-five yields aligns with G-CSF mobilization biology, in which peripheral blood CD34+ counts rise progressively to a peak between day four and day five before declining [11,12]. The median day-five to day-four ratio of 1.42 and within-donor median difference of 1.54 × 10^6^ CD34+ cells/kg sit within the range of paired and longitudinal analyses, where day-five counts have been reported as approximately 1.6- to 1.8-fold higher than day-four counts in healthy allogeneic donors [13], and the direction held at the stricter four and five million per kilogram thresholds. Series reporting equivalent day-four and day-five yields have relied on unpaired comparisons, including a multicenter Italian cohort of 840 donors in which 81.97% of day-four and 81.62% of day-five collections reached the four million per kilogram target with no significant difference and a German single-center experience with median final product CD34+ content of 8.0 × 10^6^/kg on day four and 9.2 × 10^6^/kg on day five [8,14]. In both, day-four donors had already been selected as adequate mobilizers and were not the same individuals collected on day five. The paired within-donor design here removes that selection effect and shows that the same donor systematically yields more on day five than on day four, even when both yields exceed the institutional threshold (Table A1). 

The day-four peripheral blood CD34+ count showed moderate discriminative performance for day-four dose adequacy, with an area under the receiver operating characteristic curve of 0.739. This sits within the predictive ceiling reported in the broader literature, where multivariable models built on routine donor and laboratory variables typically explain only 19–26% of yield variance [6,7]. At the institutional threshold of 20 cells/µL, the rule operated with 96.6% sensitivity and 19.4% specificity, capturing nearly all donors capable of an adequate single-session collection, while triaging a substantial fraction to day four who would fall short of the target. This profile reflects deliberate design. Falsely deferring an adequate mobilizer to day five costs an additional day of G-CSF exposure, whereas falsely clearing an inadequate mobilizer for day-four collection costs a second apheresis session, and the institutional rule has been calibrated to the former. Other centers have adopted higher thresholds, including 50 cells/µL, where peripheral blood CD34+ below this cutoff has been associated with 12.8-fold higher odds of low harvest [4,5], and 100 cells/µL for predicting very good mobilization defined as 8 × 10^6^ CD34+/kg or above, where the threshold delivers 90% sensitivity and 79% specificity [9]. These higher thresholds shift the operating point toward specificity at the cost of deferring donors from whom adequate samples could have been collected on day four. The 20 cells/µL threshold reflects the opposite priority, weighing the avoidance of mobilization failure above the avoidance of multi-session collection. In this cohort, the threshold that maximized Youden’s J statistic was 46 cells/µL (Figure 3B), more than double the institutional 20 cells/µL cutoff; because it sits higher on the CD34+ distribution, this threshold would increase specificity and reduce futile day-four collections at the expense of sensitivity, deferring a subset of borderline day-four donors who would nonetheless have achieved an adequate single-session yield.

Stratified analysis showed that day-five collection narrowed the dose adequacy gap between donors above and below the institutional threshold, with adequacy proportions converging across strata once the cumulative day-four plus day-five yield was considered. This pattern aligns with the wider literature, in which day five reliably delivers higher peripheral blood CD34+ counts than day four, and 81.1% of donors in a prior Italian cohort exceeded the same 20 cells/µL threshold on day four [15]. Formula-based predictions of day-five yield from day-four parameters have shown adjusted R^2^ values of approximately 0.71 [16,17]. reinforcing the role of day-four variables as triggers for pre-emptive intervention rather than definitive predictors of single-session sufficiency. Heterogeneity across the day-four versus day-five literature partly reflects the apheresis platform, since continuous-flow systems and the elutriation chamber design of the Spectra Optia deliver higher collection efficiencies than older devices and partly compensate for lower circulating CD34+ counts on day four [18,19]. The forty-four donors were deferred from day four because their morning count fell below the threshold reached an adequate day-five dose at a proportion comparable to the day-five yield in paired donors (Supplementary Table S2), indicating that deferral does not compromise engraftment dose in donors selected by the existing rule.

The most clinically actionable finding concerns the disposition of the collected products. Among the day-four products in donors who proceeded to a second collection, 19.1% were ultimately discarded, fresh or after cryopreservation, compared with 8.6% in donors who succeeded in a single session. This gap quantifies the downstream cost of triage decisions and has not been previously reported. A donor triaged to day-four collection on a borderline morning CD34+ count, who then proceeded to day five because the day-four yield is inadequate, typically contributes two procedures and a product that may never reach a recipient. The operational stakes of this trade-off have risen with the post-pandemic shift toward higher target doses, since the proportion of allogeneic grafts subjected to cryopreservation rose from less than 2% before March 2020 to over 80% during the early pandemic, and several centers now request five rather than four million CD34+ cells per kilogram to buffer against thaw-related viability loss [4,5]. Under-investigated baseline predictors, including ferritin and pre-mobilization peripheral blood CD34+ counts, may further refine donor selection in this stricter operational context [20]. The discard fraction reported here represents real donor effort and real institutional resources that did not translate into recipient benefit, and quantifying it is the first step toward refining the triage rule, whether by raising the day-four threshold, adopting a formula-based prediction [16], or reserving day-four collection for donors with an unambiguously high morning count. In addition, all donors received a single, fixed twice-daily filgrastim regimen; peripheral blood CD34+ mobilization kinetics and thus the optimal day-four versus day-five decision may differ with alternative mobilizing agents or doses, such as once-daily or higher-dose G-CSF or pegfilgrastim [11,12], so the present findings should not be extrapolated to those schedules.

The strengths of this study include the within-donor paired design, which minimized inter-donor variability, and the operational evaluation of an institutional day-four triage strategy with clinically relevant sensitivity analyses. However, the findings should be interpreted in light of the retrospective single-center study design, which may limit generalizability to other centers.

**Figure 4:**
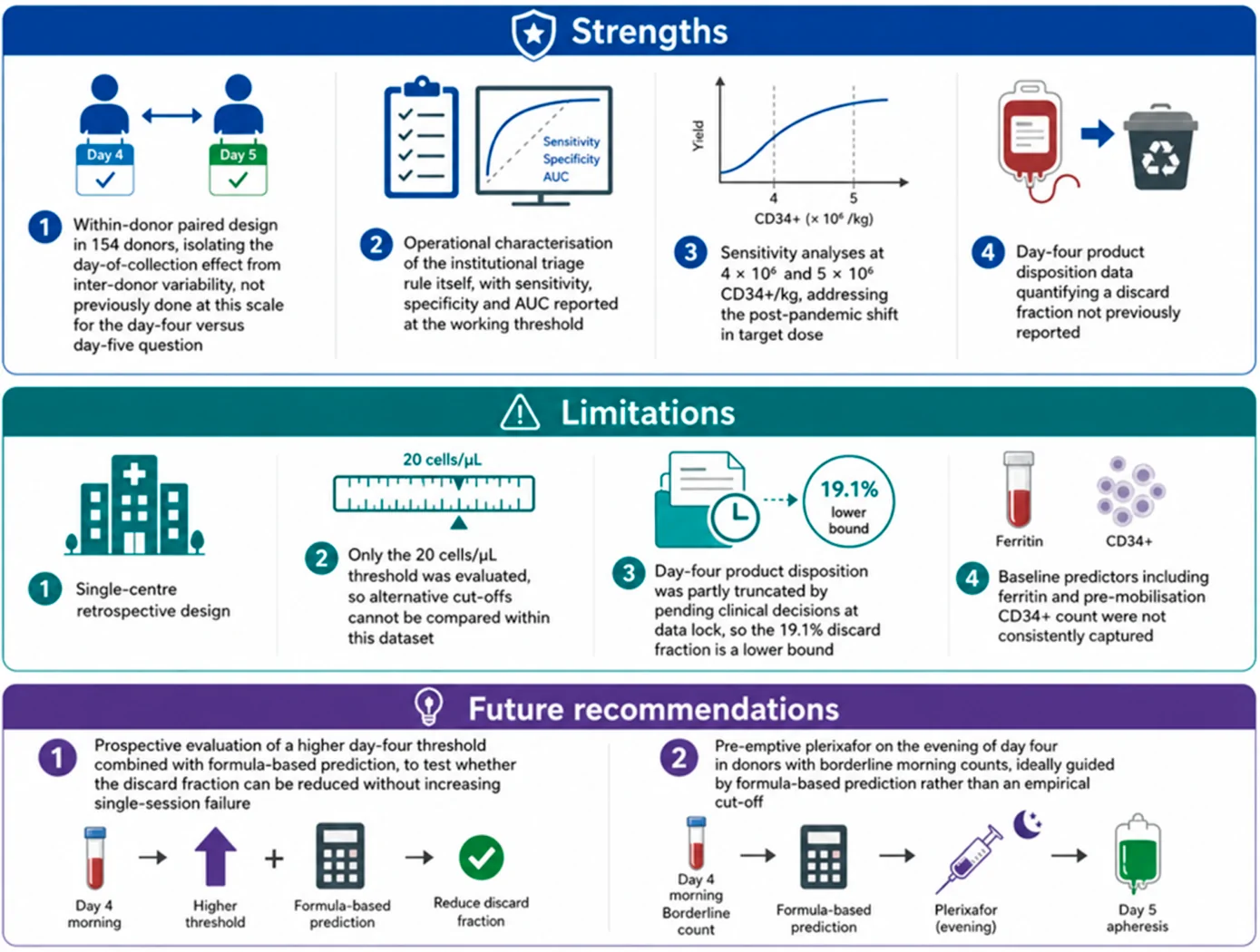
Strengths, limitations, and future directions of the analysis.

Future prospective multicenter studies incorporating recipient outcomes are needed to validate these findings and optimize day-four decision-making strategies (Figure 4).

Although our study did not perform a formal economic analysis, the findings highlight potential areas for future cost-effectiveness evaluation. The occurrence of inadequate day-four collections may represent avoidable resource utilization, including additional mobilization days, apheresis procedures, and product processing. Future studies should develop cost-effectiveness models to assess whether strategies such as a higher day-four CD34+ threshold or formula-guided pre-emptive plerixafor administration can improve resource utilization while maintaining successful transplantation outcomes.

## 5. Conclusions

The day-four CD34+ triage rule at 20 cells/µL identified nearly all donors capable of an adequate single-session collection, but with low specificity, sending a substantial fraction to a day-four apheresis that fell short of the target and produced a bag that was ultimately discarded. Within the same donor, day-five yield systematically exceeded day-four yield, and this difference was maintained at stricter dose thresholds. Refining the rule, whether by raising the threshold, adopting formula-based prediction, or reserving day-four collection for donors with an unambiguously high morning count, may reduce the discard fraction without compromising mobilization success.

## Figures and Tables

**Table 1:** Baseline characteristics of mobilization episodes by cohort group.

Variable	Group A: Day 4 Only	Group B: Paired Day 4 + 5	Group C: Day 5 Only
n (episodes)	326	156	44
Day-4 PB CD34+ (cells/µL), median (IQR)	75.7 (54.9–102.9)	47.5 (30.8–69.3)	—
Day-5 PB CD34+ (cells/µL), median (IQR)	—	75.1 (53.4–106.7)	75.9 (44.9–113.7)
Day-4 PB WBC (×10^6^/mL), median (IQR)	44.3 (37.1–54.8)	40.0 (31.6–48.3)	—
Day-4 PB platelets (×10^3^/µL), median (IQR)	241.0 (199.0–285.0)	244.0 (202.5–287.5)	—
Day-4 dose (×10^6^ CD34+/kg), median (IQR)	9.7 (7.3–13.6)	4.0 (2.6–5.5)	—
Day-5 dose (×10^6^ CD34+/kg), median (IQR)	—	5.8 (4.1–7.2)	7.1 (4.8–10.2)
Apheresis protocol, n (%)			
CMNC	56 (17.2%)	46 (29.5%)	17 (38.6%)
MNC	268 (82.2%)	109 (69.9%)	27 (61.4%)
Age, years, median (range)	-	23 (2–67)	-
Male sex, %	-	50.9	-
Female sex, %	-	49.1	-

IQR, interquartile range. PB, peripheral blood. WBC, white blood cell count. Group A: donors collected on day 4 only. Group B: donors collected on both day 4 and day 5. Group C: donors deferred and collected on day 5 only. Kruskal–Wallis test, day-4 PB CD34+ across groups: *p* ≤ 0.001.

**Table 2:** Comparison of CD34+ yield and dose adequacy between day 4 and day 5 in donors who underwent paired collections (Group B, n = 154).

Metric	Value
Median day-4 collected dose (×10^6^ CD34+/kg)	3.96 (IQR 2.64–5.44)
Median day-5 collected dose (×10^6^ CD34+/kg)	5.64 (IQR 4.06–7.16)
Median within-donor difference (day 5 − day 4)	1.54 × 10^6^/kg
95% confidence interval for difference	1.12 to 1.98
Adequate dose (≥2.5 × 10^6^/kg) on day 4 alone	118/154 (76.6%)
Adequate dose (≥2.5 × 10^6^/kg) on day 5 alone	147/154 (95.5%)
Adequate dose with combined day 4 + day 5	150/154 (97.4%)
Wilcoxon signed-rank *p*-value (day-4 vs. day-5 dose)	<0.001
McNemar test *p*-value (day-4 vs. day-5 adequacy)	<0.001

IQR, interquartile range. Adequate dose defined as ≥2.5 × 10^6^ CD34+ cells/kg recipient body weight.

**Table 3:** Engraftment dose adequacy stratified by morning-of-day-4 circulating CD34+ count in paired donors (Group B).

PB CD34+ Stratum	n	Day-4 Dose Median	Day-5 Dose Median	Day-4 Adequate, n (%)	Day-5 Adequate, n (%)
<20	11	1.98	4.87	4 (36.4%)	9 (81.8%)
≥20	144	4.24	5.80	115 (79.9%)	138 (96.5%)

PB CD34+: peripheral blood circulating CD34+ cell count on the morning of day 4, in cells/µL. Dose median: median CD34+ cells collected, expressed as ×10^6^ per kg of recipient body weight. Adequate dose: ≥2.5 × 10^6^ CD34+ cells/kg of recipient body weight. Fisher’s exact test: day-4 adequacy by stratum *p* = 0.003; day-5 adequacy by stratum *p* = 0.08.

## Data Availability

The original contributions presented in this study are included in the article/Supplementary Material. Further inquiries can be directed to the corresponding author.

## Supplementary Materials

The following supporting information can be downloaded at: https://www.mdpi.com/article/10.3390/biomedicines14081824/s1, Figure S1: Product outcome summary and detailed product fate; Table S1: Sensitivity analysis of day-4 versus day-5 dose adequacy in paired donors (Group B, n = 154) at three increasingly strict engraftment dose thresholds. Table S2. Outcomes for Group C (deferred donors, n = 44). These donors were deferred from day 4 by the existing institutional rule (PB CD34+ < 20 cells/μL) and collected on day 5 only. Their outcomes provide supportive evidence that deferral does not compromise engraftment dose: the proportion achieving an adequate dose on day 5 alone in this group is comparable to the corresponding day-5 alone result in paired donors (Group B). Table S3. Fate of collected stem cell products, stratified by cohort group and collection day.

## Appendix A

**Table A1:** Operational characteristics of allogeneic donor mobilization studies published since 2015 that report data relevant to day-four versus day-five collection or to peripheral blood CD34+ thresholds at the day of apheresis.

Study (Year)	N Donors	Setting	Comparison	D4 PB CD34+ Threshold	D5 vs. D4 Yield	D5 Alone Adequacy	Key Finding
Flommersfeld et al. 2015 [14]	100	Single-center, Germany	D4 vs. D5	Not used	D5 ~1.3 × D4	Not reported	D4 feasible with cMNC; comparable engraftment
Martino et al. 2019 [15]	122	Single-center, Italy	D4 only	≥20 cells/µL	Not applicable	Not reported	81.1% achieve ≥ 20 cells/µL on D4
Kimura et al. 2020 [13]	109	Single-center, Japan	D4 vs. D5	Not used	D4 ≈ D5; both > D6	Not separately reported	D4 yield equivalent to D5; supports earlier collection
Al-Geithi et al. 2020 [11]	165	Single-center, KSA	D4 vs. D5	Not used	D5 superior median dose	Not separately reported	D5 yields higher CD34+ doses than D4
Kayser et al. 2023 [5]	588	Single-center, Germany	D5 only	≥50 cells/µL (good mobiliser)	Not applicable	Not reported	CD34+ < 50/µL → 12.8× odds of low harvest
Passeri et al. 2023 [8]	338	Multicenter, Italy	D4 vs. D5	Not used	D5 higher; no increase in 2nd collection	~93% on D5 alone (≥4 ×10^6^/kg)	Day timing did not change second-collection rate
Yucel et al. 2024 [9]	200	Single-center, Türkiye	D4 only	≥100 cells/µL	D4 feasible if PB CD34+ ≥ 100	Not reported	PB CD34+ ≥ 100 supports single-day D4 collection
Tan et al. 2025 [16]	77	Single-center, Singapore	D4 → D5 prediction	Continuous predictor	D4 PB-CD34+ predicts D5 dose	Not reported	Pre-emptive D4 measurement enables D5 yield prediction
Present study	526	Single-center, Jordan	D4 vs. D5 (paired)	≥20 cells/µL (institutional)	D5 ~1.46 × D4 (5.78 vs. 3.96 × 10^6^/kg)	94.9% on D5 alone (≥2.5 × 10^6^/kg)	PB CD34+ ≥ 20 on D4 separates good from poor mobilisers; D5 alone adequate for 95%
