# Supplementary material for: Optimizing Timing of Peripheral Blood Stem Cell Collection: A Comparative Study of Day-4 Versus Day-5 Post-G-CSF Mobilization in Healthy Allogeneic Donors
Source: Biomedicines. 2026 Aug 13;14(8):1824. doi: 10.3390/biomedicines14081824 (PMC13509721; doi:10.3390/biomedicines14081824)
Supplement: Supplementary file 1 [file biomedicines-14-01824-s001.zip › Supplementary_Table_S1_sensitivity.docx.pdf]

Supplementary Table S1. Sensitivity analysis of day-4 versus day-5 dose adequacy in paired donors (Group B, n = 154) at three increasingly strict engraftment dose thresholds.

Threshold	Day 4 alone adequate	Day 5 alone adequate	Combined adequate	McNemar p-value
≥ 2.5 × 10^6/kg	118 / 154 (76.6%)	147 / 154 (95.5%)	150 / 154 (97.4%)	<0.001
≥ 4.0 × 10^6/kg	76 / 154 (49.4%)	118 / 154 (76.6%)	127 / 154 (82.5%)	<0.001
≥ 5.0 × 10^6/kg	47 / 154 (30.5%)	89 / 154 (57.8%)	100 / 154 (64.9%)	<0.001

Adequate dose was defined at three sequential thresholds. The 2.5 × 10^6/kg threshold corresponds to the institutional protocol; 4 × 10^6/kg corresponds to the EBMT recommendation for hematological diseases; 5 × 10^6/kg has been adopted by some centers post-COVID for cryopreservation buffer.
