# Supplementary material for: Optimizing Timing of Peripheral Blood Stem Cell Collection: A Comparative Study of Day-4 Versus Day-5 Post-G-CSF Mobilization in Healthy Allogeneic Donors
Source: Biomedicines. 2026 Aug 13;14(8):1824. doi: 10.3390/biomedicines14081824 (PMC13509721; doi:10.3390/biomedicines14081824)
Supplement: Supplementary file 1 [file biomedicines-14-01824-s001.zip › Supplementary_Table_S2_groupC.docx.pdf]

Supplementary Table S2. Outcomes for Group C (deferred donors, n = 44). These donors were deferred from day 4 by the existing institutional rule (PB CD34+ < 20 cells/μL) and collected on day 5 only. Their outcomes provide supportive evidence that deferral does not compromise engraftment dose: the proportion achieving an adequate dose on day 5 alone in this group is comparable to the corresponding day-5 alone result in paired donors (Group B).

Metric	Value
n (episodes)	44
Day-5 dose (×10^6 CD34+/kg), median (IQR)	7.1 (4.8–10.2)
Adequate dose (≥ 2.5 × 10^6/kg) on day 5 alone, n (%)	42 (95.5%)
Episode total dose (×10^6 CD34+/kg), median (IQR)	7.1 (4.8–10.2)

Group C: donors deferred from day 4 because day-4 PB CD34+ was below 20 cells/μL, then collected on day 5 only.
