# Supplementary material for: Optimizing Timing of Peripheral Blood Stem Cell Collection: A Comparative Study of Day-4 Versus Day-5 Post-G-CSF Mobilization in Healthy Allogeneic Donors
Source: Biomedicines. 2026 Aug 13;14(8):1824. doi: 10.3390/biomedicines14081824 (PMC13509721; doi:10.3390/biomedicines14081824)
Supplement: Supplementary file 1 [file biomedicines-14-01824-s001.zip › Supplementary_Table_S3_product_fate.docx.pdf]

Supplementary Table S3. Fate of collected stem cell products, stratified by cohort group and collection day.

Collection day	Product fate	Group A: Day 4 only	Group B: Paired	Group C: Day 5 only
Day 4	Infused fresh (full)	218 (66.9%)	85 (54.5%)	—
	Leftover frozen	66 (20.2%)	8 (5.1%)	—
	Pending	13 (4.0%)	41 (26.3%)	—
	Discarded fresh	20 (6.1%)	21 (13.5%)	—
	Leftover discarded	8 (2.5%)	1 (0.6%)	—
	Unknown	1 (0.3%)	—	—
Day 5	Infused fresh (full)	—	118 (75.6%)	31 (70.5%)
	Leftover frozen	—	8 (5.1%)	5 (11.4%)
	Pending	—	15 (9.6%)	5 (11.4%)
	Discarded fresh	—	12 (7.7%)	2 (4.5%)
	Leftover discarded	—	3 (1.9%)	1 (2.3%)
	Unknown	—	—	—

Percentages are within column (i.e., among collections of that day in that group).

Pending: patient had not yet been transplanted at data lock.

Discarded fresh: product discarded without freezing.

Leftover frozen: residual cells from a clinical infusion, cryopreserved.

Leftover discarded: cryopreserved residual cells subsequently discarded.
